# Symmetrical and asymmetrical insights into student adoption of AI-enhanced digital educational systems

**DOI:** 10.3389/fpsyg.2026.1970854

**Published:** 2026-10-29

**Authors:** Zhang Xiaotong, Liao Xin, Zhou Qing

**Affiliations:** 1 Department of Building Energy and Environment, Southwest Jiaotong University Hope College, Chengdu, China; 2 Railway Engineering Department, Sichuan University of Architectural Technology, Deyang, China; 3 School of Business, Southwest Jiaotong University Hope College, Chengdu, China

**Keywords:** AI-enhanced educational systems, behavioral intention, PLS-ANN-NCA, symmetrical and asymmetrical insights, technology acceptance

## Abstract

**Background:**

Based on the stimulus–organism–response (SOR) model and the technology acceptance model (TAM), this study investigates how the functional and experiential features of artificial intelligence (AI)-enhanced educational systems influence users’ behavioral intention through perceived usefulness and perceived ease of use.

**Methods:**

A multi-method approach integrating partial least squares structural equation modelling (PLS-SEM), artificial neural networks (ANN) and necessary condition analysis (NCA) was employed.

**Results:**

The results of the PLS-SEM analysis indicate that both perceived usefulness and perceived ease of use have a significant positive impact on behavioral intention, with perceived ease of use exerting the strongest effect. Furthermore, the effects of functional and experiential features on perceived usefulness and perceived ease of use are asymmetric. ANN analysis confirms the robustness of the findings and identifies perceived ease of use as the most influential determinant of behavioral intention. In contrast, the NCA analysis indicates that perceived usefulness is the only statistically significant necessary condition for achieving higher levels of behavioral intention. Although this necessary relationship is statistically significant, its effect size is small, suggesting that its actual constraining effect is relatively limited. The bottleneck analysis further reveals the constraining characteristics of different stages in the formation of behavioral intention.

**Discussion:**

By integrating symmetric, asymmetric and necessity-based analytical perspectives, this study provides a more comprehensive understanding of user acceptance behavior in AI-enhanced educational environments.

## 1 Introduction

The rapid development of artificial intelligence (AI) technology has transformed its role in the education system from a supplementary tool to an embedded foundational learning system (Chen et al., 2020; Liu et al., 2021). AI-enhanced digital education systems are continuously reshaping the way students acquire knowledge and engage in learning through features such as learning analytics, adaptive recommendations and intelligent interaction (Chen et al., 2020). Existing research indicates that a large number of universities worldwide have already adopted AI-driven learning systems to varying degrees to support personalized learning and instructional decision-making (Liu et al., 2021; Sajja et al., 2025). Zhang et al. (2025) notes that China is systematically advancing the deep integration of AI into the education system, opening up new avenues through digitalization to build a high-quality educational landscape. China is also piloting and promoting AI education centers across provinces, cities and universities (Khanal et al., 2025; Liu et al., 2026a; Zhang et al., 2025). This trend indicates that AI is shifting from a supplementary technology to the core of the learning environment, exerting a profound influence on students’ learning behaviors and decision-making processes. Poquet and De Laat (2021) argues that the integration of AI systems into learning processes at universities offers particularly significant benefits for college students, who are adults with independent learning capabilities. However, some scholars contend that embedding AI into educational systems is a double-edged sword; excessive reliance on AI may lead to mental inertia and even a decline in students’ abilities (Liu et al., 2025; Qian and Xie, 2026; Zhang et al., 2025). Against this backdrop, understanding how students evaluate and adopt AI-based educational systems has become a critical issue in the fields of educational technology and information systems.

Although the application of AI technology in higher education institutions has deepened, significant variations remain in students’ use and adoption of these technologies (Chen et al., 2020; Maheshwari, 2024; Wang et al., 2021). Existing research generally indicates that the personalized recommendations, intelligent analysis and real-time feedback capabilities of AI systems help enhance learning efficiency and the overall learning experience (Poquet and De Laat, 2021; Sajja et al., 2025). However, these technological features may also lead to cognitive load, technological uncertainty and trust issues, thereby undermining students’ willingness to use them (Zhai et al., 2024). The coexistence of technological empowerment and resistance to adoption suggests that the impact of AI-based educational systems on student behavior is not monolithic but rather operates through multidimensional and complex mechanisms (Liu et al., 2021; Poquet and De Laat, 2021; Sajja et al., 2025). More importantly, AI systems are not homogeneous technologies; they comprise various functional and experiential features, and different features may play distinct roles in shaping students’ cognition and behavior (Sajja et al., 2025; Zhai et al., 2024). This issue has yet to be systematically analyzed in the existing literature.

Existing research primarily relies on the technology acceptance model (TAM) to explain students’ usage behavior towards digital learning systems, emphasizing the central role of perceived usefulness and perceived ease of use in the formation of behavioral intentions (Unal and Uzun, 2021; Zhai et al., 2024). However, TAM’s explanatory power in AI contexts remains limited. The model focuses primarily on individual cognitive evaluations and treats technology as a relatively homogeneous, instrumental object, making it difficult to capture the complex technological environment of AI systems, which comprise multidimensional functional and experiential characteristics (FakhrHosseini et al., 2024). In contrast, the stimulus–organism–response (SOR) theory emphasizes that external environmental stimuli trigger behavioral responses through an individual’s internal psychological states (Lin et al., 2023; Wang et al., 2024), thereby providing a more contextually rich theoretical framework for understanding the impact of technological environments on user behavior. In AI-enhanced digital education systems, the system’s functional and experiential features can be regarded as key stimuli, while perceived usefulness and perceived ease of use constitute the core cognitive responses that, in turn, influence students’ behavioral intentions. However, the separate application of TAM and SOR theories often limits an in-depth understanding of the complex mechanisms underlying AI educational systems, making it difficult to reveal how multidimensional technological features jointly influence student behavior through cognitive pathways.

Furthermore, existing research generally relies on symmetry-based analytical methods such as structural equation modelling, which implicitly assume that variables have linear and interchangeable relationships. In the complex context of AI educational systems, this approach may be overly simplistic. The influence of different system characteristics on behavioral intentions may exhibit nonlinear relationships, and their effects may not increase uniformly (Al-Mamary, 2025; Ma et al., 2024; Wang et al., 2021). Certain key variables may be necessary conditions for achieving high levels of behavioral intention, meaning that when these variables do not reach a minimum threshold, improvements in other variables cannot compensate for their absence. This relationship involving necessity constraints and asymmetry is often difficult to identify within the framework of traditional linear analysis (Cao et al., 2026; Loh et al., 2022). Relying solely on a single method may lead to an underestimation of the actual role played by key variables in the process of behavior formation, thereby limiting the interpretative depth of research conclusions.

Given the aforementioned theoretical and methodological shortcomings and research gaps, this paper integrates the SOR and TAM theories to construct an analytical framework for the use behavior of AI-enhanced digital education systems. Within this framework, the system’s functional characteristics (personalization, intelligence and content completeness) and experiential characteristics (interactivity, anthropomorphism and responsiveness) are treated as external stimuli that influence students’ perceived usefulness and perceived ease of use, thereby affecting their behavioral intentions. At the same time, this study adopts a multi-method approach integrating partial least squares structural equation modelling (PLS-SEM), artificial neural networks (ANN) and necessary condition analysis (NCA) to reveal the mechanisms of interaction among variables from both symmetric and asymmetric perspectives. This study first examines the linear relationships among system characteristics, cognitive variables and behavioral intentions; second, it identifies the relative importance of each variable in predicting behavioral intentions; and finally, it explores the necessary conditions and threshold levels required to achieve high behavioral intentions.

## 2 Theoretical foundation and hypothesis development

### 2.1 Linear symmetrical relationship

#### 2.1.1 Perceived usefulness, perceived ease of use and behavior intention

TAM, proposed by Davis (1989), emphasizes the central role of perceived usefulness and perceived ease of use in technology adoption, viewing user behavior as the result of rational cognitive evaluations. However, this model implicitly treats technological systems as relatively homogeneous instrumental objects, thereby overlooking the structural differences inherent in the technological environment itself. In the context of AI technology, this assumption has certain limitations. This is because AI systems are typically composed of multidimensional functionalities and interactive features, and different features may influence users’ cognition and behavioral intentions through distinct pathways. In contrast, the SOR theory emphasizes the influence of external environmental stimuli on an individual’s internal psychological state, which in turn affects behavioral responses (Lin et al., 2023; Wang et al., 2024). In AI educational systems, the system’s multidimensional features can be regarded as key stimuli (S), while users’ cognitive evaluations (R) constitute the organism-level responses (O).

In the TAM, perceived usefulness is regarded as a core factor influencing users’ behavioral intentions; it reflects an individual’s subjective evaluation of a technology’s ability to enhance task performance (FakhrHosseini et al., 2024; Papakostas et al., 2023; Trivedi et al., 2022). Murtaza et al. (2022) suggests that, in the context of AI-based educational systems, students are more inclined to use systems that effectively support learning objectives and improve learning efficiency. Compared with traditional information systems, AI systems can provide students with more targeted learning support through data analysis and intelligent recommendations, thereby reinforcing their instrumental value (Liu et al., 2021; Sajja et al., 2025; Unal and Uzun, 2021). It is worth noting that, in AI contexts, perceived usefulness not only stems from the functionality itself but may also be influenced by algorithmic transparency and the credibility of results, making its underlying mechanisms more complex (FakhrHosseini et al., 2024; Glassberg et al., 2025; Grimmelikhuijsen, 2023). Nevertheless, existing research generally indicates that when users perceive a system as useful, their willingness to engage with it increases significantly (Papakostas et al., 2023; Shahzad et al., 2024). Therefore, the following hypothesis is proposed:

*H1*: Perceived usefulness has a significant positive influence on behavioral intention.

Meanwhile, Trivedi et al. (2022) emphasized that perceived ease of use precisely reflects the degree of difficulty users experience when operating a system. In AI-based educational systems, as system capabilities continue to be enhanced and updated, their complexity may also increase, thereby raising the learning curve for users (Murtaza et al., 2022; Suryanarayana et al., 2024). If a system’s interface is overly complex or its interaction logic is unclear, users may be less inclined to use it because of usability barriers, even if they recognize its functional value. From the SOR perspective, negative cognitive reactions at the organism level weaken the formation of behavioral responses (Lin et al., 2023; Wang et al., 2024). Specifically, a high level of difficulty in use often triggers negative cognitive evaluations, which in turn inhibit users’ behavioral intentions. Therefore, whether a system is easy to understand and operate remains a critical factor influencing students’ usage decisions. Therefore, the following hypothesis is proposed:

*H2*: Perceived ease of use has a significant positive influence on behavioral intention.

#### 2.1.2 Functional features, perceived usefulness and perceived ease of use

With the widespread adoption of AI technology, researchers have shifted their focus from whether users adopt AI systems to exploring which system features promote user adoption (Agrawal et al., 2024). Against this backdrop, functional features are regarded as key antecedent variables influencing user cognition and behavior. Existing research has defined functional features in terms of system capabilities and task support, emphasizing the role of technology in enhancing efficiency, optimizing decision-making and supporting individual needs (Gupta et al., 2022; Poquet and De Laat, 2021; Wang et al., 2021). In AI-based educational systems, Costley (2022) emphasizes that functional features are a core factor influencing students’ cognitive evaluations, as they determine the system’s ability to support learning tasks, enhance efficiency and reduce operational burden. These features not only provide technical utility but also influence behavioral intentions by stimulating users’ cognitive responses.

Gao and Liu (2023) points out that personalization is a key feature of AI systems, which analyze users’ behavioral data and preferences to provide customized content and learning pathways. Based on the SOR framework, personalization serves as a key motivating factor that enhances the alignment between learning content and individual needs, thereby increasing the system’s practical value. When learning resources are better tailored to individual needs, students are more likely to perceive the system’s positive impact on their academic performance. At the same time, personalized recommendations can reduce the effort required to filter information, enabling users to access the content they need more efficiently, thereby reducing cognitive load and enhancing the perceived ease of use of the system (Chang and Dao, 2025; Murtaza et al., 2022). However, excessive personalization may also lead to the information filter bubble effect (Areeb et al., 2023; Jindal and Gouri, 2024), a potential risk that has not yet been fully addressed in the existing literature. Therefore, the following hypothesis is proposed:

*H3*: Personalization has a significant positive influence on (a) perceived usefulness and (b) perceived ease of use.

An AI system’s capabilities in data processing and decision support are key indicators of its level of intelligence (Ma et al., 2024; Shwedeh, 2024; Zhang et al., 2025). For example, AI-powered educational systems can automatically recommend learning pathways and provide study recommendations. Within the SOR framework, intelligence not only enhances a system’s ability to support learning tasks but also optimizes the cognitive experience by reducing the decision-making burden on users (Vafaei-Zadeh et al., 2025; Wang et al., 2024). This implies that when a system can proactively identify learning needs and provide effective recommendations, students are more likely to perceive it as highly valuable. At the same time, intelligent features can streamline user workflows by automatically handling complex tasks in the background, thereby enhancing perceived ease of use. Therefore, the following hypothesis is proposed:

*H4*: Intelligence has a significant positive influence on (a) perceived usefulness and (b) perceived ease of use.

The comprehensiveness and organization of the resources provided by a system reflect its level of content integrity (Ou et al., 2026). In AI-based educational systems, content serves not only as a vehicle for information but also as a crucial component of the learning experience (Costley, 2022; Wang et al., 2021). Content with a high level of completeness reduces the need for students to switch between different platforms, thereby improving learning efficiency and enhancing the system’s usefulness. Vorm and Combs (2022) found that, in intelligent systems, content with a clear structure and coherent logic helps reduce comprehension difficulty, thereby improving the system’s ease of use. However, excessive content or an unclear structure may also increase cognitive load, suggesting that the relationship between content completeness and user experience is not necessarily linear. Therefore, the following hypothesis is proposed:

*H5*: Content completeness has a significant positive influence on (a) perceived usefulness and (b) perceived ease of use.

#### 2.1.3 Experience features, perceived usefulness and perceived ease of use

In Cheng et al. (2023) research, the authors examined how experiential characteristics during the interaction process influence user behavior. These experiential characteristics emphasize the perceived quality of user–system interaction, including interaction methods, feedback mechanisms and contextual immersion. From an SOR perspective, the experiential characteristics of AI-based educational systems indirectly influence students’ cognitive evaluations by optimizing the interaction process and enhancing the engagement experience, thereby further affecting their behavioral intentions (Vafaei-Zadeh et al., 2025; Wang et al., 2024; Yadav et al., 2025). System interactivity reflects the degree of information exchange between students and the system (Al-Sayid and Kirkil, 2023). Sabri et al. (2024) found that a high level of interactivity enhances students’ sense of engagement in learning, allowing them to receive immediate feedback during use and thereby deepening their understanding of the learning content. Based on SOR theory, dynamic feedback during interaction can serve as an environmental stimulus, activating students’ cognitive engagement and consequently improving their evaluation of the system’s usefulness (Vafaei-Zadeh et al., 2025; Wang et al., 2024; Zhou et al., 2023). At the same time, Niu et al. (2025) emphasizes that smooth and intuitive interaction design can reduce the complexity of system operation, thereby enhancing perceived ease of use. However, some studies offer a different perspective, suggesting that overly complex or frequent interactions may increase the operational burden, leading to cognitive fatigue and thereby undermining the system’s positive effects (Al-Sayid and Kirkil, 2023; García-Barrios, 2025; Niu et al., 2025; Wei et al., 2026). Therefore, the following hypothesis is proposed:

*H6*: Interactivity has a significant positive influence on (a) perceived usefulness and (b) perceived ease of use.

With the advancement of AI, AI systems are increasingly exhibiting human-like characteristics during interactions (Chandra et al., 2022; Siemon et al., 2026), such as conversational customer service, voice feedback or emotionally charged prompts. Xia et al. (2026) argues that, in AI-based educational systems, anthropomorphic design can enhance users’ sense of social presence, creating a more natural interactive experience for students. These human-like features help reduce the psychological distance associated with technology use, thereby improving perceived ease of use (Jiang and Xu, 2026; Liu et al., 2026b; Xia et al., 2026). In addition, they can strengthen emotional connections and trust, indirectly enhancing perceived usefulness. However, existing studies also suggest that anthropomorphism may trigger discomfort or the uncanny valley effect, particularly when a system’s interaction design does not align with user expectations. This indicates that the effects of anthropomorphism are not uniformly positive and require a balanced approach to design and implementation. Therefore, the following hypothesis is proposed:

*H7*: Anthropomorphism has a significant positive influence on (a) perceived usefulness and (b) perceived ease of use.

The speed and accuracy with which a system responds to user input reflect its responsiveness (Li and Gu, 2026; Zhang et al., 2024). In AI-based educational systems, rapid and accurate feedback can effectively reduce waiting time and uncertainty, thereby enhancing users’ sense of control and satisfaction (Costley, 2022; Ou et al., 2026; Zhang et al., 2024). Existing research indicates that high responsiveness, as a key stimulus, helps reinforce students’ cognitive experiences and further enhances their perceived usefulness and perceived ease of use (Chen et al., 2025; FakhrHosseini et al., 2024; Papakostas et al., 2023; Vorm and Combs, 2022), which is consistent with the mechanism proposed by SOR theory. Wang and Lo (2025) showed that response delays and error messages significantly reduce user trust and negatively affect usage intention, indicating that responsiveness plays a critical role in experience design. Therefore, the following hypothesis is proposed:

*H8*: Responsiveness has a significant positive influence on (a) perceived usefulness and (b) perceived ease of use.

### 2.2 Asymmetrical relationship

Although previous studies have primarily examined the impact of the functional characteristics (personalization, intelligence and content completeness), experiential characteristics (interactivity, anthropomorphism and responsiveness), perceived ease of use and perceived usefulness of AI-based educational systems on students’ behavioral intentions from a linear and symmetrical perspective (Chen et al., 2025), this single paradigm has limitations in the context of AI-based educational systems. The process through which students use educational systems is not a simple linear sequence linking cognitive evaluations to behavioral intentions; rather, it is a dynamic system (García-Barrios, 2025; Lin et al., 2023; Loh et al., 2022; Osei and Bjorklund, 2024). Shahzad et al. (2024) argues that various system characteristics, cognitive perceptions and behavioral intentions interact and co-evolve within AI-based educational systems. For example, highly responsive experiences or engaging content may rapidly trigger students’ perceptions of usefulness; even when the levels of personalization or intelligence are relatively low, behavioral intentions may still increase as a result.

In this context, functional characteristics, experiential characteristics, perceived usefulness and perceived ease of use do not occur sequentially or accumulate in a simple manner; rather, they operate in parallel. For example, a lack of personalization may limit perceived usefulness and perceived ease of use, but high-quality interactivity can immediately enhance students’ cognitive evaluations and behavioral intentions. These interactive and potentially contradictory effects suggest that the formation of students’ behavioral intentions follows a nonlinear and asymmetric logic rather than a unidirectional, linear cumulative process (Cao et al., 2026; Gao and Liu, 2023; García-Barrios, 2025; Wang et al., 2024). Traditional symmetric analytical methods can effectively test the average linear relationships between predictor and outcome variables (Greco et al., 2025; Hait et al., 2025). However, in the heterogeneous context of AI educational systems, the emergence of high behavioral intention is often contingent upon the presence of certain key system characteristics (Costley, 2022; Loh et al., 2022; Wang et al., 2024). When these characteristics fall below a minimum threshold, high behavioral intention is unlikely to be observed. In this context, NCA provides a valuable complement, as it identifies which characteristics serve as indispensable prerequisites for the development of high behavioral intention (Dul, 2024a; Richter et al., 2020; Sukhov et al., 2022).

Based on the aforementioned nonlinear and asymmetric perspectives, this study employs an integrated analytical framework comprising PLS-SEM, ANN and NCA, with the aim of simultaneously explaining both the average mechanisms (linear and symmetric effects) and the constraint structures (nonlinear and asymmetric effects) underlying students’ willingness to use AI-enhanced educational systems.

In summary, we propose a conceptual framework (see Figure 1).

**Figure 1:**
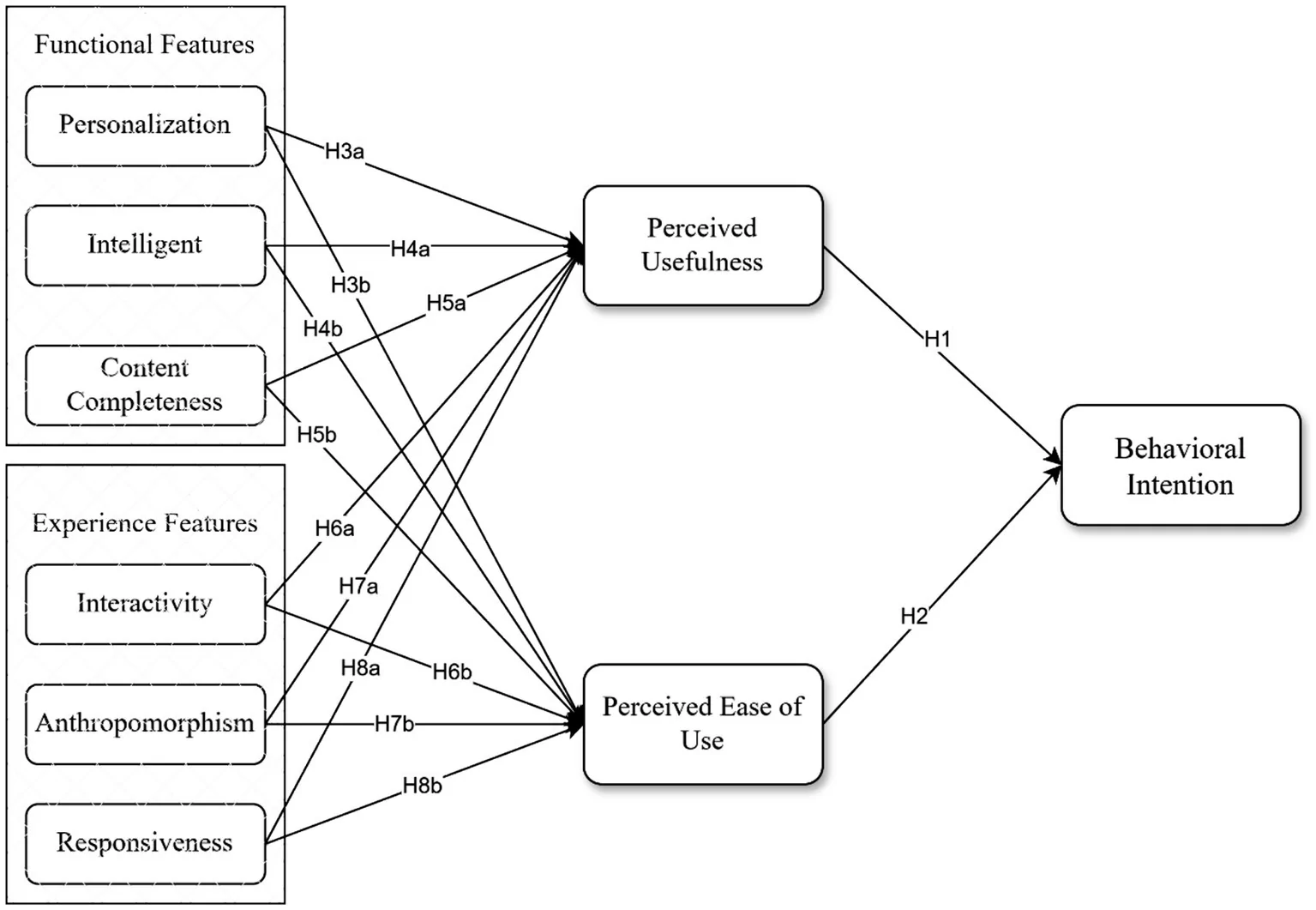
Conceptual framework.

## 3 Method

### 3.1 Sample and procedure

This study focuses on students at Chinese universities who had used AI-powered educational systems for learning within the previous 12 months. To ensure that the research objectives were clear and well defined, respondents were provided with a brief explanation of the study’s purpose and conceptual framework. Online questionnaires were distributed through channels such as university faculty members and mainstream social media platforms, and data collection took place between November and December 2025. During the data collection process, strict operational protocols were followed to ensure participant anonymity, data confidentiality and secure data storage. Although the study focused on a sample of university students in China, this group is highly representative in terms of exposure to AI applications and AI-based educational systems (Ma et al., 2024; Zhang et al., 2025). It effectively reflects students’ usage patterns and behavioral intentions within the context of AI educational systems.

Participants were recruited using a combination of convenience and snowball sampling. Given that users of AI-enabled digital education systems are geographically dispersed, convenience sampling can effectively reach college students who meet the study criteria, thereby improving the efficiency of questionnaire distribution and data collection. At the same time, combining this with snowball sampling—where respondents recommend other students with relevant experience—helps expand the sample coverage and increase the response rate among the target population. All respondents were university students with experience using AI-based educational systems, enabling them to provide reliable data for this study. By including two screening questions (‘Have you used an AI-based educational system for learning in the past 12 months?’—for example, Chinese AI-based educational systems such as iFlytek Spark, ERNIE Bot, Xueersi Intelligent Teaching Assistant, Zuoyebang AI Teacher, and intelligent question bank systems—and ‘Are you currently enrolled in a higher education institution?’), we ensured that only eligible respondents participated in the survey. After excluding 19 incomplete questionnaires, a total of 301 valid responses were retained for data analysis.

The sample size for this study was calculated using G*Power software. The G*Power analysis indicated that a minimum sample size of 160 participants was required to detect a significant effect. According to the guidelines of Nuchitprasittichai and Cremaschi's (2013), the validation sample size for an ANN model should be at least 10 times the number of independent variables; thus, a minimum sample size of 80 cases was required to ensure sufficient statistical power and support robust analysis. Furthermore, unlike methods based on linearity or symmetry, NCA does not priorities statistical power when determining sample size; rather, it emphasizes whether the sample adequately covers the possible range of values for the condition and outcome variables (Dul, 2024a; Richter et al., 2020). Therefore, the final sample size of 301 participants exceeded the minimum requirements for the aforementioned analytical methods.

### 3.2 Research design

This study employed a quantitative research approach with a cross-sectional questionnaire survey. PLS-SEM was used to test the proposed hypotheses and examine the relationships among the constructs. ANN analysis was subsequently conducted to assess the relative importance of the significant predictors, while NCA was used to identify the necessary conditions for achieving high levels of behavioral intention. These methods were used to examine the research questions from explanatory, predictive, and necessity perspectives.

### 3.3 Measures

A structured questionnaire was developed based on the research objectives and relevant literature, and a pilot test was conducted to assess the clarity and suitability of the questionnaire before necessary revisions were made. The questionnaire scales were primarily based on established and well-validated instruments and were appropriately adapted to align with the context of AI educational systems, thereby ensuring a high degree of fit between the measurement content and the research context, as well as strong content validity. All measurement items were assessed using a 7-point Likert scale, ranging from 1 (strongly disagree) to 7 (strongly agree). Personalization was measured using an adapted version of the scale developed by Bleier and Eisenbeiss (2015), while intelligence was assessed using an adapted version of the Gursoy et al. (2019) and Park (1988) scale. Content completeness was measured using a modified version of the Ballou and Pazer (2003) scale, and interactivity was assessed using items adapted from the Fan et al. (2017) scale. Anthropomorphism was measured using the Gursoy et al. (2019) scale, while responsiveness and perceived usefulness were assessed using adapted versions of the Gunness et al. (2023) scale. The items measuring perceived ease of use and behavioral intention were adapted from the Venkatesh et al. (2003) study.

To ensure the face validity and content validity of the scale, this study invited three educational experts to evaluate the questionnaire items and revised certain wording based on their feedback. The questionnaire was initially developed in Chinese and subsequently translated into English using a two-way translation process, in which two researchers with relevant expertise independently performed the translation and back-translation, followed by multiple rounds of comparison to ensure semantic consistency (Benlidayi and Gupta, 2024). The final questionnaire was presented in English to enhance its international readability. The results of a pilot study conducted prior to the main survey indicated that some items exhibited low internal consistency. Therefore, only those items demonstrating satisfactory reliability and validity were retained for the subsequent survey.

## 4 Data analysis

### 4.1 Stages of statistical analysis

This study employed SmartPLS 4.0 to conduct PLS-SEM analysis, followed by SPSS 27.0 for ANN analysis, and finally used the R programming language to perform NCA. These three analytical methods were utilized to systematically investigate the impact of AI-enhanced educational systems on university students’ behavioral intentions. PLS-SEM is a variance-based structural equation modelling technique that is suitable for testing causal relationships among latent variables, as well as path effects and linear relationships within complex models. ANN is primarily used to capture nonlinear relationships among variables, thereby enhancing predictive power and identifying key influencing factors. NCA is used to identify the prerequisites necessary to achieve a given outcome, emphasizing the minimum threshold levels of variables, their constraining effects on the outcome, and the nonlinear nature of these prerequisite conditions.

### 4.2 Demographic profile

Based on the demographic profile presented in Table 1, the gender distribution was relatively balanced, with males accounting for 47.5% of the sample. The largest age groups were concentrated in the age brackets of18–24 and 25–34 years, representing 33.6% (101 participants) and 35.5% (107 participants), respectively. More than 55% of the students were enrolled in associate and bachelor’s degree program.

**Table 1:** Demographic profile.

Characteristic	Categories	Frequency	Percent (%)
Gender	Male	143	47.5
	Female	158	52.5
Age	18–24	101	33.6
	25–34	107	35.5
	35–44	87	28.9
	Above 45	6	2.0
Education	Associate degree	137	45.5
	Bachelor’s degree	91	30.2
	Master’s degree	51	16.9
	PhD	22	7.3

### 4.3 Results of PLS-SEM analysis

#### 4.3.1 Measurement model evaluation

Table 2 presents the reliability and validity results for the scales used in this study. The factor loadings for all items exceeded 0.7, indicating that each subscale effectively reflected its underlying construct (Baharum et al., 2023). The Cronbach’s α and composite reliability (CR) values for all constructs were above 0.7, while the average variance extracted (AVE) values exceeded 0.5, indicating good internal consistency and convergent validity (Hair et al., 2017). To assess multicollinearity, variance inflation factor (VIF) values were examined, and all values were below 5, indicating that there were no serious multicollinearity issues (Hair et al., 2017; Purwanto, 2021). Additionally, this study employed the heterotrait–monotrait ratio (HTMT) to assess discriminant validity (see Table 3). The results showed that all HTMT values were below 1, indicating good discriminant validity among the constructs (Voorhees et al., 2016). To assess common method bias (CMB), Harman single factor test was conducted. The first factor explained 31.815% of the total variance, below the 50% threshold, indicating no serious common method bias (Hair et al., 2017).

**Table 2:** Measurement model.

Construct	Factor loading	Mean	Std. deviation	Cronbach’s *α*	CR	AVE
Personalization (PERS)				0.920	0.922	0.807
PERS1	0.886	5.73	1.465			
PERS2	0.905	5.60	1.594			
PERS3	0.913	5.48	1.696			
PERS4	0.888	5.51	1.582			
Intelligent features (INTEL)				0.935	0.940	0.837
INTEL1	0.906	5.29	1.797			
INTEL2	0.916	5.27	1.785			
INTEL3	0.911	5.23	1.894			
INTEL4	0.927	5.48	1.712			
Content completeness (CONT)				0.921	0.922	0.808
CONT1	0.900	5.49	1.646			
CONT2	0.907	5.57	1.467			
CONT3	0.901	5.51	1.574			
CONT4	0.887	5.63	1.641			
Interactivity (INTR)				0.952	0.952	0.838
INTR1	0.917	5.40	1.789			
INTR2	0.912	5.31	1.855			
INTR3	0.927	5.15	1.871			
INTR4	0.911	5.32	1.857			
INTR5	0.910	5.34	1.783			
Anthropomorphism (ANTH)				0.939	0.944	0.805
ANTH1	0.900	5.57	1.689			
ANTH2	0.924	5.43	1.685			
ANTH3	0.900	5.63	1.627			
ANTH4	0.893	5.47	1.708			
ANTH5	0.869	5.54	1.626			
Responsiveness (RESP)				0.939	0.941	0.803
RESP1	0.910	5.52	1.696			
RESP2	0.895	5.63	1.558			
RESP3	0.902	5.56	1.655			
RESP4	0.889	5.55	1.694			
RESP5	0.885	5.59	1.571			
Perceived usefulness (PU)				0.940	0.945	0.807
PU1	0.898	5.38	1.764			
PU2	0.889	5.43	1.734			
PU3	0.906	5.25	1.795			
PU4	0.903	5.38	1.868			
PU5	0.895	5.41	1.723			
Perceived ease of use (PEOU)				0.917	0.918	0.801
PEOU1	0.872	5.47	1.710			
PEOU2	0.888	5.50	1.612			
PEOU3	0.912	5.39	1.732			
PEOU4	0.907	5.55	1.613			
Behavioral intention (BI)				0.941	0.941	0.808
BI1	0.911	5.57	1.655			
BI2	0.900	5.61	1.557			
BI3	0.903	5.52	1.593			
BI4	0.899	5.61	1.643			
BI5	0.882	5.61	1.536			

**Table 3:** HTMT.

	ANTH	BI	CONT	INTEL	INTR	PEOU	PERS	PU	RESP
ANTH									
BI	0.347								
CONT	0.368	0.409							
INTEL	0.298	0.290	0.317						
INTR	0.213	0.243	0.326	0.270					
PEOU	0.317	0.459	0.408	0.401	0.282				
PERS	0.296	0.394	0.419	0.377	0.353	0.352			
PU	0.249	0.396	0.324	0.220	0.344	0.348	0.399		
RESP	0.325	0.363	0.305	0.356	0.317	0.375	0.346	0.356	

### 4.4 Hypothesis testing: linear symmetrical relationship (H1–H8)

After evaluating the measurement model, this study employed PLS-SEM to test the research hypotheses. The structural model was used to examine the path relationships among the functional characteristics (personalisation, intelligence and content completeness), experiential characteristics (interactivity, anthropomorphism and responsiveness), perceived usefulness, perceived ease of use and behavioral intention.

Table 4 shows that the path coefficients and significance levels were estimated using 5,000 bootstrap samples. The results indicate that both perceived usefulness (PU) and perceived ease of use (PEOU) have significant positive effects on behavioural intention (BI). Specifically, the effect of PU on BI was significant (*β* = 0.265, *p* = 0.000), and the effect of PEOU on BI was also significant and stronger (*β* = 0.340, *p* = 0.000), thereby supporting H1 and H2. These findings suggest that, in the context of AI-enhanced educational systems, students are more inclined to adopt systems that are both useful and easy to use, with perceived ease of use playing a more critical role in the formation of behavioral intention.

**Table 4:** Structure model.

Paths specified	Standardized coefficient	*p*-value	Finding
H1: PU → BI	0.265	0.000***	Supported
H2: PEOU → BI	0.340	0.000***	Supported
H3a: PERS → PU	0.197	0.001**	Supported
H3b: PERS → PEOU	0.090	0.091n.s.	Not supported
H4a: INTEL → PU	−0.037	0.464n.s.	Not supported
H4b: INTEL → PEOU	0.192	0.000***	Supported
H5a: CONT → PU	0.080	0.164n.s.	Not supported
H5b: CONT → PEOU	0.188	0.002**	Supported
H6a: INTR → PU	0.156	0.009**	Supported
H6b: INTR → PEOU	0.062	0.296n.s.	Not supported
H7a: ANTH → PU	0.047	0.362n.s.	Not supported
H7b: ANTH → PEOU	0.093	0.083n.s.	Not supported
H8a: RESP → PU	0.155	0.015*	Supported
H8b: RESP → PEOU	0.155	0.002**	Supported
SRMR composite model = 0.033			
R^2^BI = 0.244	Q^2^BI = 0.196		
R^2^PEOU = 0.269	Q^2^PEOU = 0.234		
R^2^PU = 0.249	Q^2^PU = 0.199		

*p < 0.05, **p < 0.01, ***p < 0.001.

In terms of functional characteristics, personalization (PERS) had a significant positive effect on perceived usefulness (PU) (*β* = 0.197, *p* = 0.001), but its effect on PEOU was not significant (*β* = 0.090, *p* = 0.091.). Therefore, H3a was supported, whereas H3b was not supported. Intelligence (INTEL) had a significant positive effect on PEOU (*β* = 0.192, *p* = 0.000), but its effect on PU was not significant (*β* = −0.037, *p* = 0.464.). Thus, H4b was supported, whereas H4a was not supported. Content completeness (CONT) had a significant positive effect on PEOU (*β* = 0.188, *p* = 0.002), but no significant effect on PU (*β* = 0.080, *p* = 0.164.). Accordingly, H5b was supported, whereas H5a was not supported.

Regarding experiential characteristics, the results also revealed differential effects. Interactivity (INTR) had a significant positive effect on PU (*β* = 0.156, *p* = 0.009), but its effect on PEOU was not significant (*β* = 0.062, *p* = 0.296.), supporting H6a but not H6b. Anthropomorphism (ANTH) did not have a significant effect on either PU or PEOU (*β* = 0.047, *p* = 0.362.; *β* = 0.093, *p* = 0.083.); therefore, neither H7a nor H7b was supported. In contrast, responsiveness (RESP) had a significant positive effect on both PU (*β* = 0.155, *p* = 0.015) and PEOU (*β* = 0.155, p 0.002), thereby supporting H8a and H8b.

### 4.5 Results of ANN

Following the completion of the PLS-SEM analysis, this study employed an ANN to assess the relative importance of the antecedent factors of the endogenous variables. Specifically, a feedforward neural network model was constructed using the SPSS platform, featuring a single hidden layer and trained using the backpropagation algorithm. The number of neurons in the hidden layer was adjusted through multiple iterations to minimize prediction error. The hidden layer employed a sigmoid activation function, while the output layer used a linear activation function; this configuration is appropriate for predicting continuous dependent variables (Kouadri et al., 2022; Nuchitprasittichai and Cremaschi, 2013; Pannakkong et al., 2022; Patil et al., 2023). Given the relatively complex structure of the overall model, the ANN analysis was divided into three sub-models to improve prediction stability and accuracy. First, with PU as the output variable, the standardized factor scores of the six antecedent variables (ANTH, CONT, INTEL, INTR, PERS and RESP) identified in the PLS-SEM phase were used as input variables. Second, with PEOU as the output variable, the input layer similarly comprised the standardized factor scores of the same six constructs. Finally, with BI as the output variable, a third model was constructed using PU and PEOU as the input variables.

This study employed a 10-fold cross-validation approach, in which 90% of the data were used for model training and the remaining 10% for model testing (Pannakkong et al., 2022). A lower root mean square error (RMSE) value indicates superior predictive performance (Kouadri et al., 2022; Nuchitprasittichai and Cremaschi, 2013). In the ANN model with PU as the output variable, the mean RMSE was 0.612 for the training set and 0.606 for the test set. In the model with PEOU as the output variable, the mean RMSE was 0.606 for the training set and 0.629 for the test set. In the model with BI as the output variable, the mean RMSE was 0.620 for the training set and 0.628 for the test set. Overall, the results presented in Table 5 indicate that the models demonstrated good predictive accuracy.

**Table 5:** Training testing.

Neural network	Training			Testing			
	*N*	SSE	RMSE	*N*	SSE	RMSE	Total (*N*)
Perceived usefulness (PU)							
ANN1	265	90.372	0.584	36	8.697	0.492	301
ANN2	268	93.874	0.592	33	8.681	0.513	301
ANN3	259	106.733	0.642	42	12.730	0.551	301
ANN4	274	93.458	0.584	27	12.133	0.670	301
ANN5	267	104.293	0.625	34	16.207	0.690	301
ANN6	264	93.618	0.595	37	11.266	0.552	301
ANN7	278	110.763	0.631	23	9.430	0.640	301
ANN8	265	110.589	0.646	36	14.760	0.640	301
ANN9	268	91.281	0.584	33	11.548	0.592	301
ANN10	270	110.409	0.639	31	16.187	0.723	301
Mean		100.539	0.612		12.164	0.606	
*SD*		8.288	0.025		2.691	0.074	
Perceived ease of use (PEOU)							
ANN1	270	94.699	0.592	31	13.773	0.667	301
ANN2	266	83.516	0.560	35	10.345	0.544	301
ANN3	268	97.445	0.603	33	18.309	0.745	301
ANN4	272	90.680	0.577	29	8.557	0.543	301
ANN5	281	120.228	0.654	20	5.948	0.545	301
ANN6	280	133.603	0.691	21	14.230	0.823	301
ANN7	263	85.214	0.569	38	5.311	0.374	301
ANN8	278	109.448	0.627	23	13.723	0.772	301
ANN9	272	97.421	0.598	29	7.616	0.512	301
ANN10	281	95.660	0.583	20	11.692	0.765	301
Mean		100.791	0.606		10.950	0.629	
*SD*		15.029	0.039		3.939	0.138	
Behavioral intention (BI)							
ANN1	273	96.970	0.596	28	8.176	0.540	301
ANN2	269	92.073	0.585	32	9.685	0.550	301
ANN3	268	95.402	0.597	33	18.652	0.752	301
ANN4	268	92.626	0.588	33	16.298	0.703	301
ANN5	267	91.957	0.587	34	8.636	0.504	301
ANN6	270	113.332	0.648	31	16.570	0.731	301
ANN7	274	122.657	0.669	27	10.058	0.610	301
ANN8	263	95.853	0.604	38	20.340	0.732	301
ANN9	274	112.226	0.640	27	4.681	0.416	301
ANN10	263	124.530	0.688	38	20.883	0.741	301
Mean		103.763	0.620		13.398	0.628	
*SD*		12.370	0.036		5.488	0.114	

Sensitivity analysis was conducted to assess the relative importance of each exogenous variable in predicting the dependent variable. The results showed that, in the PU model, PERS was the most influential factor (100%), followed by RESP (74.40%), INTR (62.70%), CONT (60.75%), ANTH (44.20%) and INTEL (35.83%). In the PEOU model, CONT had the greatest influence (100%), followed by INTEL (74.22%), RESP (67.98%), PERS (66.20%), ANTH (60.36%) and INTR (42.99%). In the BI model, PEOU was the most influential predictor (100%), followed by PU (84.70%). Finally, the results of the ANN analysis were compared with those of the PLS-SEM analysis to assess the consistency between the linear and nonlinear methods. As shown in Table 6, the two methods yielded consistent and highly aligned results across four pathways (H1, H2, H4b and H6b).

**Table 6:** Sensitivity analysis.

Neural network	ANTH	CONT	INTEL	INTR	PERS	RESP
Perceived usefulness (PU)						
ANN1	0.250	1.000	0.566	0.962	0.953	0.487
ANN2	0.584	1.000	0.431	0.663	0.837	0.827
ANN3	0.533	0.935	0.269	0.759	1.000	0.759
ANN4	0.368	0.293	0.347	0.441	1.000	0.592
ANN5	0.542	0.698	0.082	0.530	1.000	0.737
ANN6	0.477	0.499	0.171	0.588	1.000	0.894
ANN7	0.379	0.140	0.711	0.459	1.000	0.992
ANN8	0.360	0.186	0.182	0.154	1.000	0.670
ANN9	0.619	0.654	0.510	0.942	1.000	0.624
ANN10	0.215	0.542	0.239	0.640	1.000	0.702
Average relative importance	0.433	0.595	0.351	0.614	0.979	0.728
Normalised relative importance (%)	44.20%	60.75%	35.83%	62.70%	100.00%	74.40%
Perceived ease of use (PEOU)						
ANN1	0.299	1.000	0.683	0.174	0.313	0.719
ANN2	0.400	1.000	0.823	0.206	0.617	0.525
ANN3	0.250	0.993	1.000	0.450	0.774	0.483
ANN4	0.397	1.000	0.335	0.132	0.374	0.301
ANN5	1.000	0.959	0.157	0.989	0.769	0.688
ANN6	1.000	0.664	0.244	0.428	0.821	0.271
ANN7	0.248	1.000	0.998	0.156	0.309	0.777
ANN8	0.655	0.364	0.489	0.640	1.000	0.594
ANN9	0.642	0.935	1.000	0.262	0.479	0.746
ANN10	0.490	1.000	0.888	0.396	0.446	0.956
Average relative importance	0.538	0.892	0.662	0.383	0.590	0.606
Normalised relative importance (%)	60.36%	100.00%	74.22%	42.99%	66.20%	67.98%

Neural network	PU	PEOU
Behavioral intention (BI)		
ANN1	0.619	1.000
ANN2	0.653	1.000
ANN3	0.981	1.000
ANN4	0.758	1.000
ANN5	0.553	1.000
ANN6	1.000	0.771
ANN7	0.463	1.000
ANN8	0.703	1.000
ANN9	1.000	0.734
ANN10	1.000	0.621
Average relative importance	0.773	0.913
Normalised relative importance (%)	84.70%	100.00%

### 4.6 Results of NCA

NCA, proposed by Dul (2024b), is a method used to identify whether a specific condition constitutes a necessary prerequisite for the occurrence of an outcome, while also measuring the strength of its influence. A necessary condition can therefore be regarded as a constraint or bottleneck factor, meaning that the target outcome can only be achieved when that condition is satisfied. Figure 2 illustrates scatter plots of the relationships among the antecedent variables, mediating variables and the dependent variable in the model used in this study. NCA determines the presence of necessary conditions by identifying empty areas within the scatter plots (Loh et al., 2022; Richter et al., 2020; Sukhov et al., 2022). A typical NCA scatter plot includes the ordinary least squares (OLS) regression line, the ceiling envelopment–free disposal hull (CE-FDH) line and the ceiling regression–free disposal hull (CR-FDH) line. The empty area in the upper-left corner of the plot reflects the constraining effect of the independent variable X on the dependent variable Y. As shown in Figure 2, a degree of empty space is present in the upper-left corner of each plot, indicating a potential constraint relationship. However, this relationship must be further verified through significance testing to determine whether it constitutes a necessary condition.

**Figure 2:**
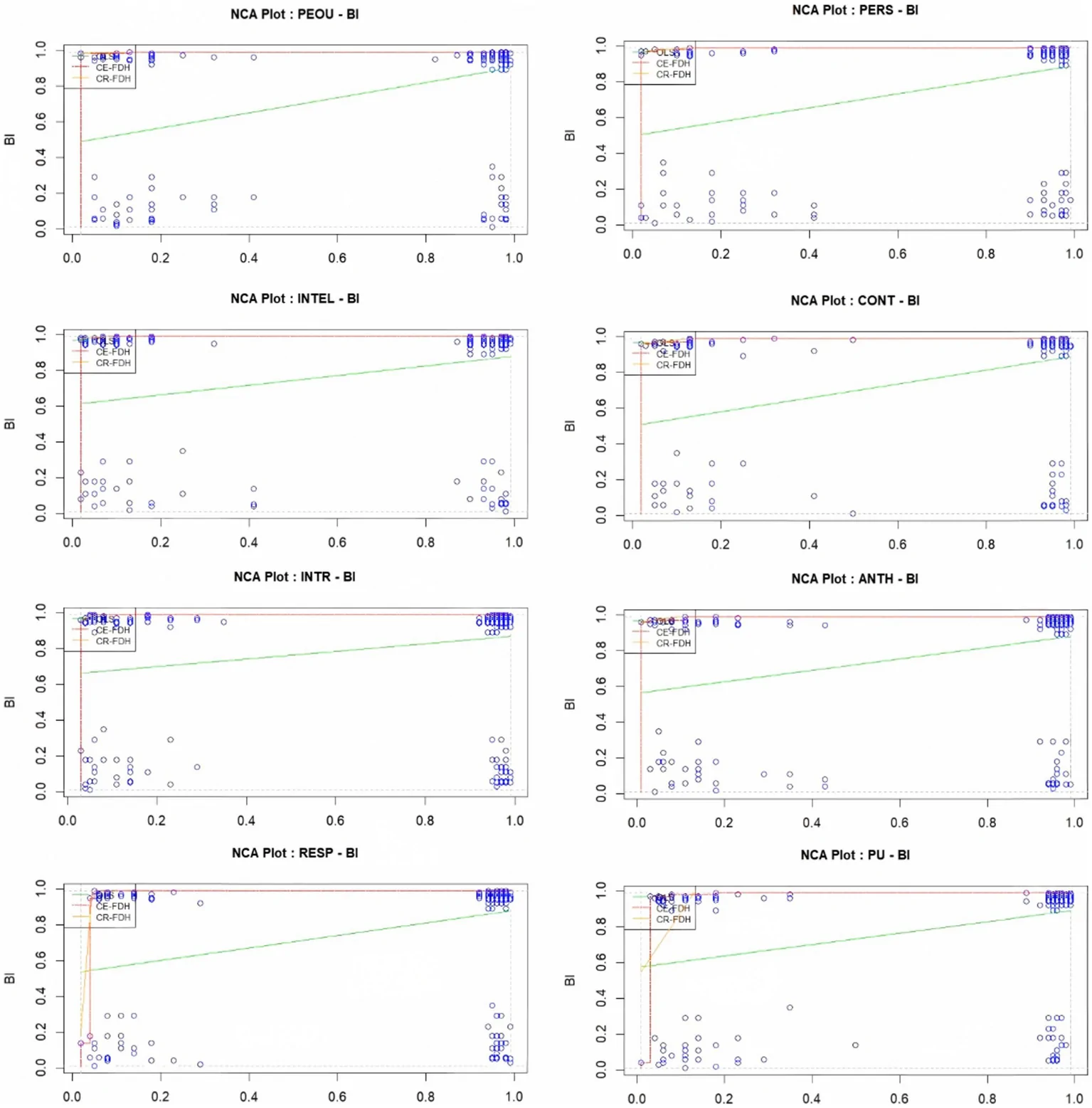
NCA plot.

Subsequently, NCA was used to calculate key metrics, including the ceiling region, accuracy, precision, coverage and effect size. To assess the validity of the ceiling line, particular attention should be paid to the accuracy and effect size parameters (Dul, 2024a; Richter et al., 2020; Sukhov et al., 2022). The effect size represents the proportion of the total space occupied by the empty region above the ceiling line and is typically calculated as the ratio of the ceiling area to the total area. This metric reflects the minimum threshold required to achieve the outcome variable. To determine whether a variable constitutes a necessary condition, three basic criteria must be met: first, it must have clear theoretical support; second, the effect size must be greater than 0; and third, the *p*-value must reach statistical significance (Dul, 2024b; Dul et al., 2023). According to the classification criteria proposed by Dul et al. (2023), an effect size between 0 and 0.1 is considered small, 0.1–0.3 moderate, 0.3–0.5 large and greater than 0.5 very large. The results presented in Table 7 indicate that the effect sizes for all variables fall within the 0–0.1 range, corresponding to small effects. Based on these results, perceived usefulness constitutes a necessary condition for behavioral intention.

**Table 7:** PLS-ANN ranking.

Path	PLS-SEM: path coefficient	ANN: normalised relative importance (%)	Ranking PLS SEM (based on path coefficient)	Ranking ANN (based on normalised relative importance, %)	Remark
H1: PU → BI	0.265	84.70%	2	2	Matched
H2: PEOU → BI	0.34	100.00%	1	1	Matched
H3a: PERS → PU	0.197	100.00%	3	1	
H3b: PERS → PEOU	0.09	66.20%	9	6	
H4a: INTEL → PU	−0.037	35.83%	13	12	
H4b: INTEL → PEOU	0.192	74.22%	4	4	Matched
H5a: CONT → PU	0.08	60.75%	10	8	
H5b: CONT → PEOU	0.188	100.00%	5	1	
H6a: INTR → PU	0.156	62.70%	6	7	
H6b: INTR → PEOU	0.062	42.99%	11	11	Matched
H7a: ANTH → PU	0.047	44.20%	12	10	
H7b: ANTH → PEOU	0.093	60.36%	8	9	
H8a: RESP → PU	0.155	74.40%	7	3	
H8b: RESP → PEOU	0.155	67.98%	7	5	

Table 8 presents a bottleneck analysis designed to assess criticality. The NCA bottleneck table shows the degree of conditionality required for different outcome levels (Dul, 2024b; Richter et al., 2020; Sukhov et al., 2022). The analysis method uses CR, while NN indicates non-criticality. The first column of the bottleneck analysis represents different outcome levels of behavioral intention, while the second and subsequent columns represent the corresponding levels of necessary conditions. These levels can be expressed as percentage ranges, actual values, or percentiles. For example, when behavioral intention reaches 30%—a relatively high outcome level—only responsiveness exhibits a bottleneck value of 0.3%, which constitutes a weak bottleneck and can also be interpreted as a soft constraint. This represents a fundamental factor in ensuring interaction. When behavioral intention reaches the intermediate outcome level (60%), responsiveness and perceived usefulness reach bottleneck values of 1.3% each. When behavioral intention reaches the highest outcome level (100%), all variables collectively constitute necessary conditions, exhibiting a multidimensional and synergistic bottleneck structure. Overall, the results indicate that the formation of behavioral intention is not driven by a single factor but instead exhibits phased bottleneck characteristics. Different variables assume distinct roles in terms of necessity at different developmental stages. Among these, responsiveness constituted a fundamental limiting factor, while perceived usefulness (PU, 12.2%) and perceived ease of use (PEOU, 11.3%) emerged as relatively strong bottlenecks in achieving the highest levels of behavioral intention. Specifically, at the highest target level of behavioral intention (BI = 100%), the bottleneck values for PU and PEOU were the two highest across all conditions, indicating that both need to reach relatively high levels to support the achievement of the highest level of behavioral intention. It is worth noting that although PU exhibited statistically significant necessity in the NCA, its necessity effect size was small, whereas the necessity effect size for PEOU was 0, suggesting that the overall degree of necessity constraint for both factors was not strong. However, from the perspective of the target level in the bottleneck analysis, when behavioral intention is elevated to the highest level, PU and PEOU still exhibit a relatively prominent constraining effect. Therefore, although the actual constraining effect of PU and PEOU is limited in terms of overall necessity, maintaining high levels of both remains crucial for achieving the highest level of behavioral intention (see Table 9).

**Table 8:** Analysis results of necessary conditions for the NCA method (CR method).

Conditions	Behavioral intention	
	Effect size (*d*)	*p*-value
ANTH	0.001	0.342
CONT	0.002	0.308
INTEL	0	0.473
INTR	0	0.369
PERS	0.001	0.255
RESP	0.01	0.145
PU	0.027	0.011*
PEOU	0	0.592

(1) Conditions refer to calibrated fuzzy membership values; (2) 0 ≤ *d* <0.1 indicates low-level effects, while 0.1 ≤ *d* <0.3 indicates medium-level effects; (3) *p < 0.05, **p < 0.01, ***p < 0.001; (4) the permutation test in NCA analysis employed 10,000 resampling iterations.

**Table 9:** Analysis results of bottleneck levels (%) using the NCA method.

BI	PERS	INTEL	CONT	INTR	ANTH	RESP	PU	PEOU
0	NN	NN	NN	NN	NN	NN	NN	NN
10	NN	NN	NN	NN	NN	NN	NN	NN
20	NN	NN	NN	NN	NN	NN	NN	NN
30	NN	NN	NN	NN	NN	0.3	NN	NN
40	NN	NN	NN	NN	NN	0.6	NN	NN
50	NN	NN	NN	NN	NN	0.9	NN	NN
60	NN	NN	NN	NN	NN	1.3	1.3	NN
70	NN	NN	NN	NN	NN	1.6	4.1	NN
80	NN	NN	NN	NN	NN	1.9	6.8	NN
90	NN	NN	NN	NN	NN	2.3	9.5	NN
100	11.1	5.2	11.2	2.2	10	2.6	12.2	11.3

(1) The analysis method is CR; (2) NN stands for “unnecessary”.

## 5 Discussion and conclusion

Drawing on the SOR and TAM frameworks, this study employed a multi-method approach incorporating PLS-SEM, ANN and NCA to explore how the functional and experiential features of AI-enhanced educational systems influence students’ behavioral intentions through cognitive evaluation. The study identifies the nuanced factors shaping students’ behavioral intentions and provides deeper insights into higher education students’ intentions to use AI-enhanced educational systems.

### 5.1 Linear symmetrical relationship

The PLS-SEM analysis revealed that both perceived usefulness (*β* = 0.265, *p* = 0.000) and perceived ease of use (*β* = 0.340, *p* = 0.000) had significant positive effects on behavioral intention, with perceived ease of use exerting a stronger effect than perceived usefulness. The perceived ease of use observed in this study had a stronger impact; this finding is consistent with the results of previous empirical studies on AI-assisted learning and digital educational technologies (Vorm and Combs, 2022). The findings suggest that this may be attributed to the relative interactivity and dynamism of AI-enhanced educational systems. In such systems, students continuously interact with AI-generated content, recommendations, and feedback. When the system is intuitive to use and requires minimal cognitive and motor effort, students may be more willing to integrate it into their learning activities.

In terms of functional characteristics, the effects of different antecedent variables on perceived usefulness and perceived ease of use differed significantly. Personalization had a significant positive effect on perceived usefulness (*β* = 0.197, *p* = 0.001) but no significant effect on perceived ease of use (*β* = 0.090, *p* = 0.091.), indicating that personalization primarily enhances users’ perceptions of the system’s value but does not significantly reduce the perceived difficulty of use. In contrast, both intelligence (*β* = 0.192, *p* = 0.000) and content completeness (*β* = 0.188, *p* = 0.002) had significant positive effects on perceived ease of use. However, neither intelligence (*β* = −0.037, *p* = 0.464.) nor content completeness (*β* = 0.080, *p* = 0.164.) had a significant effect on perceived usefulness. This finding is consistent to some extent with previous empirical studies, which have shown that personalization primarily enhances users’ perception of utility by tailoring content and recommendations to individual needs (Chen et al., 2025). However, the finding that there is no significant association between personalization and perceived ease of use differs from the conclusions of studies that suggest personalized interfaces can simplify users’ interactions with digital systems (Gunness et al., 2023). One possible explanation is that, in AI-enhanced educational systems, personalization primarily involves the type of content or learning support provided, whereas perceived ease of use focuses on the convenience of user interaction with the system. Therefore, personalization may enhance functional value but does not necessarily reduce the operational effort required of students.

In terms of experience characteristics, the research findings also revealed differing effects. Interactivity significantly enhanced perceived usefulness (*β* = 0.156, *p* = 0.009) but had no significant effect on perceived ease of use (*β* = 0.062, *p* = 0.296.). This suggests that the interactive process can enhance users’ perception of the system’s value but cannot effectively reduce perceived complexity. This finding is consistent with previous empirical research, which indicates that interactivity can enhance users’ perception of a system’s value by facilitating real-time communication, feedback, and positive engagement with digital systems (Gao and Liu, 2023). However, the finding that it had no significant effect on perceived ease of use (*β* = 0.062, *p* = 0.296.) suggests that higher interactivity does not necessarily imply a reduction in effort required for use. In AI-enhanced educational systems, interactive features may provide richer learning support and facilitate more timely interactions, thereby increasing perceived utility; however, the additional interactions and information generated during the learning process do not necessarily make the system easier to operate. Anthropomorphism did not have a significant effect on either perceived usefulness (*β* = 0.047, *p* = 0.362.) or perceived ease of use (*β* = 0.093, *p* = 0.083.). Previous research has shown that anthropomorphism significantly influences user behavior; however, the findings of this study differ from those earlier empirical results (Liu et al., 2026a; Siemon et al., 2026; Xia et al., 2026). This finding suggests that users pay relatively little attention to anthropomorphic features, or that the anthropomorphic design may not yet have reached a level capable of significantly influencing user perceptions.

### 5.2 Nonlinear asymmetrical relationship

Since functional and experiential characteristics do not simultaneously influence perceived usefulness and perceived ease of use, but instead affect user cognition through distinct pathways, they exhibit an asymmetric interaction pattern. The ANN results showed that the RMSE values for both the training and test sets were low and relatively similar, indicating good predictive stability and generalizability. In terms of variable importance, significant differences were observed across the different dependent variables. In the perceived usefulness model, personalization was the most influential predictor, followed by responsiveness and interactivity. In contrast, content completeness and intelligence played dominant roles in the perceived ease of use model. In the behavioral intention model, perceived ease of use was more influential than perceived usefulness. These findings further indicate that the effects of different variables on the outcome variables are not evenly distributed but instead exhibit distinct pathways and varying levels of influence. Such differences may arise from users placing different levels of emphasis on specific system features during cognitive processing, thereby creating a divergence between value judgements and assessments of usage effort. Furthermore, the ANN and PLS-SEM results demonstrated a high degree of consistency across key pathways (H1, H2, H4b and H6b), indicating that, although the two methods are based on nonlinear and linear assumptions, respectively, they show strong stability in identifying core relationships. This consistency helps mitigate the interpretive risks associated with methodological bias and suggests that the study’s findings are robust.

The NCA results indicate that perceived usefulness is the only significant necessary condition for the formation of behavioral intention, whereas perceived ease of use, despite exhibiting the strongest influence in both the PLS-SEM and ANN analyses, does not constitute a necessary condition. This finding highlights the fundamental distinction between influence and necessity: perceived ease of use is a key driver of behavioral intent, whereas the necessity of perceived usefulness is primarily statistically significant but remains relatively limited in practical terms. In other words, even if a system is easy to use, users are unlikely to develop a strong behavioral intent unless they believe it offers genuine value.

The NCA bottleneck analysis further revealed the stage-specific characteristics of behavioral intention formation. The results showed that, at lower levels of behavioral intention, responsiveness alone constituted a weak bottleneck, reflecting its role as a foundational factor in facilitating user interaction. As behavioral intention increased to a moderate level, the perceived usefulness associated with responsiveness began to exert a constraining effect. At higher levels of behavioral intention, all variables collectively constituted necessary conditions, forming a multidimensional and synergistic bottleneck structure. In particular, at the highest level of behavioral intention, the threshold values for perceived usefulness and perceived ease of use were the highest, highlighting their critical roles in achieving strong behavioral intentions. Overall, the formation of behavioral intention is not driven by a single factor. Rather, different variables assume greater importance at different stages of development, reflecting the dynamic and hierarchical nature of the process.

### 5.3 Theoretical contribution

By integrating the SOR and TAM frameworks, this study develops a multi-path explanatory framework and extends the theoretical boundaries of traditional technology acceptance research. Whereas previous TAM studies have emphasized the direct effects of perceived usefulness and perceived ease of use on behavioral intention, this study introduces multidimensional system characteristics as antecedent variables, thereby revealing how different types of stimuli influence cognitive evaluations through distinct pathways. This integration not only enriches the conceptual framework of the SOR model but also transforms TAM from a single-cognition model into a more comprehensive framework driven by multiple sources of stimulation.

Second, this study makes an important theoretical contribution by revealing the asymmetry of variable effects and the differences among influence pathways. The results indicate that different system characteristics do not simultaneously affect perceived usefulness and perceived ease of use; rather, they exhibit a selective pattern of influence. This finding challenges the assumption, common in much of the existing literature, that antecedent variables exert a universal effect on both perceived usefulness and perceived ease of use. The study demonstrates that user cognition is not homogeneous but is shaped by the degree of alignment between different types of stimuli and specific cognitive dimensions, thereby exhibiting complex structural characteristics.

Third, by introducing ANN and NCA methods, this study advances methodological development in the field of technology acceptance. Traditional SEM is primarily used to test linear relationships between variables, whereas ANN can identify nonlinear patterns and assess the relative importance of variables, and NCA reveals the ‘missing-at-entry’ constraint mechanism from the perspective of necessary conditions. The combination of these three methods enables the study not only to determine whether an effect exists, but also to explain which factors are more important and what is missing to achieve the desired outcome, thereby providing a more multidimensional interpretive framework for understanding user behavior.

Finally, this study proposes an analytical framework based on a phased bottleneck mechanism, emphasizing that users’ behavioral intentions are not formed in a single instance but are gradually shaped by different variables that exert constraining effects at various developmental stages. This finding offers a new theoretical entry point for future research: understanding technology adoption behavior from a dynamic and process-oriented perspective, rather than relying solely on static relational models.

### 5.4 Practical contribution

From a system design perspective, this study indicates that the formation of users’ behavioral intentions is influenced by both usability and practical value, but the three analytical methods reveal that these two factors operate in different ways. Both PLS-SEM and ANN analyses indicate that perceived ease of use has a strong influence on and predictive power for behavioral intent. Therefore, developers should prioritize optimizing core user experiences related to ease of use—such as enhancing the simplicity of the user interface, the clarity of operational workflows, and the intuitiveness of feature access—to reduce users’ learning costs and usage burdens by minimizing operational steps, optimizing navigation structures, and providing clear guides for feature usage. At the same time, NCA, as a supplementary analysis, revealed a different mechanism of action compared to the impact analysis: while perceived ease of use did not reach the necessary level of statistical significance in the NCA, this does not imply that it is unimportant. Rather, it indicates that, compared to perceived ease of use, perceived usefulness plays a more clearly necessary role in achieving higher levels of behavioral intention. Therefore, there is no substantive contradiction between PEOU and PU; rather, they represent, respectively, a strong influencing factor driving the increase in behavioral intention and a necessary constraint for achieving a higher level of behavioral intention. Based on this complementary relationship, system designers should prioritize improving usability and user experience while ensuring that the system provides accurate, comprehensive content and functions highly relevant to learning tasks, enabling users to consistently perceive its practical learning value. This approach fosters a system design strategy that balances usability optimization with the assurance of utility value.

Second, given the differences in variable importance revealed by the ANN results, system optimization should adopt a module-by-module improvement strategy rather than allocating resources evenly. For example, to enhance the system’s perceived value, features such as AI-powered personalized recommendations and customized learning paths can be strengthened to better align the system with users’ needs. To improve ease of use, the focus should be on optimizing the presentation of information and the structure of content. This can be achieved through modular content design, one-stop information integration and intelligent search functions, thereby reducing the effort users expend in searching for information. These targeted functional improvements will help to enhance the overall user experience more effectively.

Third, the results of the NCA and bottleneck analysis indicate that improvements in users’ behavioral intentions exhibit distinct phased characteristics; therefore, system design should adopt a dynamic, phased strategy. During the initial user engagement phase, priority should be given to ensuring system responsiveness and a strong foundational user experience. For example, user trust can be established through fast loading times and immediate feedback. Once users enter a stable usage phase, system functionality and content depth can be gradually enhanced to increase users’ perception of system value. In the later, advanced usage phase, multidimensional and coordinated optimization (such as feature refinement, user experience improvements and content upgrades) is necessary to maintain users’ willingness to continue using the system.

Fourth, from a system operations perspective, the research findings underscore the fundamental importance of responsiveness; therefore, platforms should establish stable and efficient feedback mechanisms. For example, optimizing server performance, improving algorithm response times and introducing real-time interactive feedback (such as instant prompts and dynamic recommendations) can ensure that users receive timely responses during their interactions. Additionally, by utilizing user behavior data monitoring systems, platforms can identify delays or interruptions in user operations in real time and implement targeted optimizations, thereby enhancing the overall user experience.

Finally, this study indicates that achieving high levels of behavioral intention depends on the synergistic interaction of multiple factors; therefore, in practice, a single-function approach should be avoided in favor of a holistic optimization mindset. For example, during the system iteration process, tools such as user experience maps can be used to identify key influencing factors at different stages and implement systematic improvements. At the same time, it is recommended to introduce user feedback mechanisms (such as periodic surveys, user interviews and usage log analysis) to continuously optimize system design, enabling it to better adapt to changing user needs.

### 5.5 Limitations and future research

Although this study has made multidimensional advances in both theory and methodology, there remains scope for further extension in future research. First, as this study relies on cross-sectional data, it is difficult to fully capture dynamic changes in user cognition and behavior over time; therefore, its ability to explain causal relationships is somewhat limited. Future research could employ a longitudinal design to dynamically track changes in user cognition and behavior across different stages of use, thereby providing deeper insights into the evolution of stage-specific bottleneck mechanisms.

Second, since the sample data is primarily based on a cohort of higher education students in a specific context, this may limit the generalizability of the findings to some extent, as results may vary across different cultural backgrounds or usage contexts. Future research could conduct cross-sample comparative studies in different cultural or educational settings to test the robustness and generalizability of the findings.

Finally, although this study employed ANN and NCA methods to enhance analytical depth, the specification of certain model parameters remains somewhat subjective; for example, the choice of neural network architecture and threshold settings in NCA may influence the results. Future research could further integrate experimental methods or behavioral data to strengthen causal inference and reduce data-driven bias.

## Data Availability

The original contributions presented in the study are included in the article/supplementary material, further inquiries can be directed to the corresponding author.
